# SGD-OD: investigating the potential oxygen demand of submarine groundwater discharge in coastal systems

**DOI:** 10.1038/s41598-024-59229-7

**Published:** 2024-04-22

**Authors:** Willard S. Moore, Claudia Benitez-Nelson, Charles Schutte, Amy Moody, Alan Shiller, Ryan J. Sibert, Samantha Joye

**Affiliations:** 1 School of the Earth, Ocean, & Environment, University of South Carolina, Columbia, SC USA; 2 Department of Environmental Science, Rowan University, Glassboro, NJ USA; 3 Division of Marine Science, Stennis Space Center, University of Southern Mississippi, Hattiesburg, MS USA; 4 Department of Marine Sciences, University of Georgia, Athens, GA USA

**Keywords:** Marine chemistry, Element cycles, Geochemistry

## Abstract

Submarine groundwater discharge (SGD) supplies nutrients, carbon, metals, and radionuclide tracers to estuarine and coastal waters. One aspect of SGD that is poorly recognized is its direct effect on dissolved oxygen (DO) demand in receiving waters, denoted here as SGD-OD. Sulfate-mediated oxidation of organic matter in salty coastal aquifers produces numerous reduced byproducts including sulfide, ammonia, dissolved organic carbon and nitrogen, methane, and reduced metals. When these byproducts are introduced to estuarine and coastal systems by SGD and are oxidized, they may substantially reduce the DO concentration in receiving waters and impact organisms living there. We consider six estuarine and coastal sites where SGD derived fluxes of reduced byproducts are well documented. Using data from these sites we present a semiquantitative model to estimate the effect of these byproducts on DO in the receiving waters. Without continued aeration with atmospheric oxygen, the study sites would have experienced periodic hypoxic conditions due to SGD-OD. The presence of H_2_S supplied by SGD could also impact organisms. This process is likely prevalent in other systems worldwide.

## Introduction

**Figure 1:**
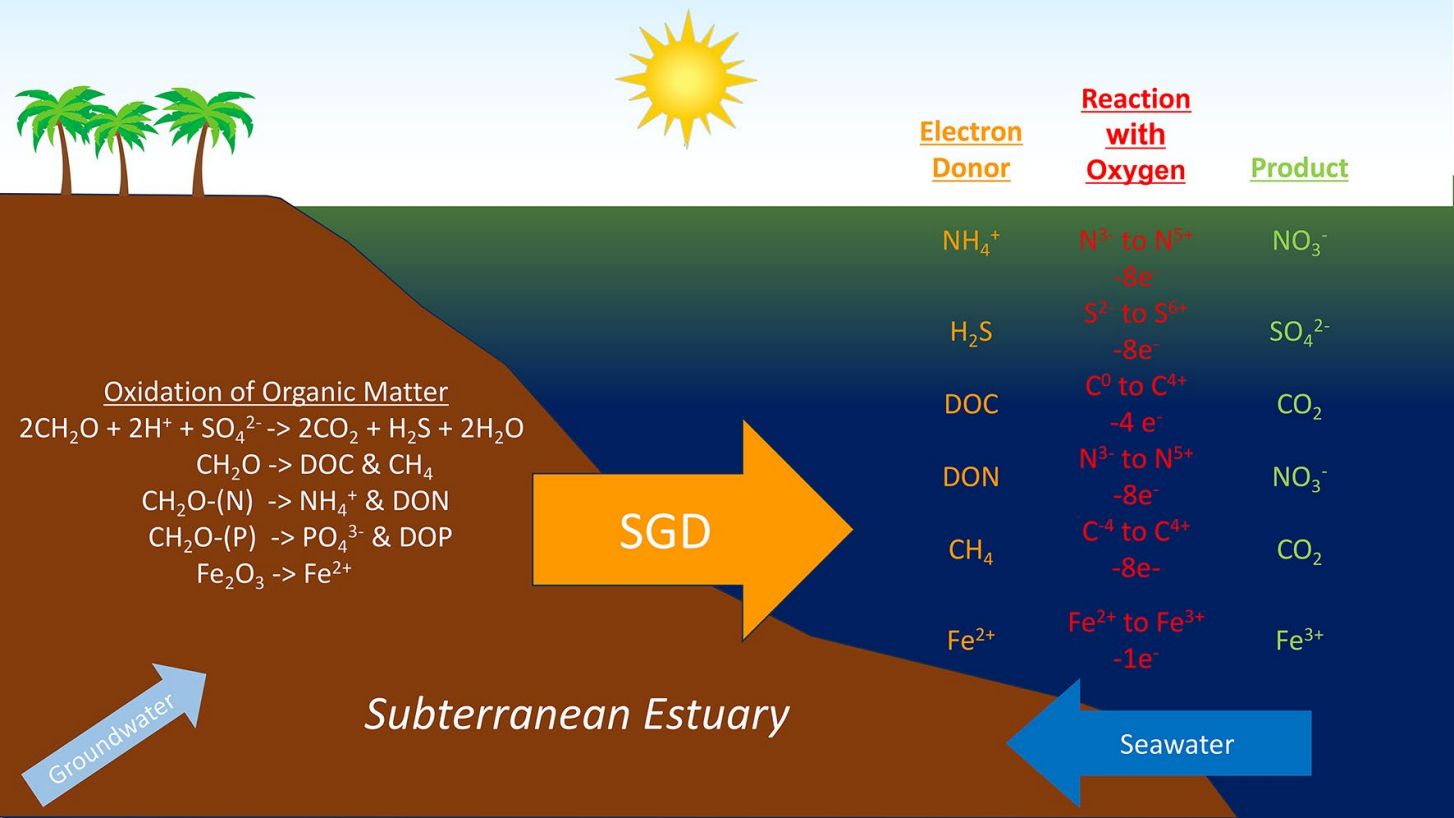
Illustration of reactions within the subterranean estuary that enrich submarine groundwater discharge (SGD) in electron donors. When these electron doners are transported to coastal waters by SGD, they react with oxygen, reducing the dissolved oxygen content of receiving waters.

Hypoxia is a widespread and growing problem in estuaries and along continental shelves across the globe^1^. It is often caused by respiration of organic matter derived from the overlying water column or upstream environments^2^. Oxygen, however, can also be consumed through the oxidation of other reduced substances, concentrations of which are often enriched in the groundwater found within coastal aquifers. These coastal aquifers can be thought of as subterranean estuaries where freshwater and seawater meet, mix, and react with sediments in the aquifer and aquiclude^3,4^. These reactions are often mediated by microorganisms^5^. The abiotic and biological reactions that occur within subterranean estuaries, especially those oxidizing organic matter (OM), enrich fluids in a suite of components, including nutrients, dissolved inorganic (DIC) and organic (DOC) carbon, sulfides (here we refer to H_2_S but recognize HS^-^ is also present) and other trace gasses (CH_4_ and N_2_O), reduced metals (Fe^2+^ and Mn^2+^), and Ra^2+^^4,6^. These reaction products dramatically alter the fluids delivered to surface estuaries and released into the coastal ocean by submarine groundwater discharge (SGD). As such, SGD components play critical roles in numerous coastal biogeochemical processes, such as biological production and community structure and as a source/sink of atmospheric CO_2_^7,8^. The reduced components in SGD also serve as electron donors in oxygen-consuming reactions that lower dissolved oxygen (DO) in coastal waters. Here, we explore the potential influence of SGD on estuarine and coastal DO concentrations and hypoxia (Fig. 1).

Oxygen is typically the dominant electron acceptor available for organic matter oxidation in meteoric (i.e., containing low total dissolved solids) groundwater, which contains about 0.28 mmol L^−1^ DO when saturated at 25 °C^4^. Saline groundwater, in contrast, contains sulfate ion (SO_4_^2-^), a strong oxidizing agent. The average concentration of SO_4_^2−^ in seawater is 28 mmol L^−1^. Thus, in groundwater systems dominated by seawater, SO_4_^2−^ may be as much as 100 times higher than the concentration of DO in freshwater^4^. In terms of oxidation capacity, each mmol of sulfate can oxidize 2 mmol of carbon [Eq. (1)], while each mmol of DO can only oxidize 1 mmol of C. Thus, the oxidation capacity of sulfate relative to DO is up to 200 times greater.

1 $${\text{2CH}}_{{2}} {\text{O }} + {\text{ 2H}}^{ + } + {\text{ SO}}_{{4}}^{{{2} - }} \to {\text{2CO}}_{{2}} + {\text{ H}}_{{2}} {\text{S}} + {\text{ 2H}}_{{2}} {\text{O}}$$

Because aquifer fluids containing sulfate can oxidize much more OM than systems with only DO, they produce more reduced byproducts, including NH_4_^+^, H_2_S, and additional DOC depending on the OC content within the aquifer. When these reduced byproducts are released by SGD, their oxidation in receiving waters drives a reduction in DO concentration. The extent of DO reduction depends on the volume of SGD, the concentrations and form of electron donors, the time scale and kinetics of the oxidation reactions, and other removal mechanisms.

This paper highlights the potential of SGD to decrease DO concentrations in estuarine and coastal waters. We use the term SGD-OD to refer to the potential oxygen demand by SGD^9^ and describe a semiquantitative technique for evaluating oxygen consumption at several sites. While the technique is based on limited data and assumptions regarding the behavior of SGD components once discharged, we nevertheless demonstrate that SGD has the potential to significantly deplete DO concentrations in multiple estuarine and coastal water systems.

## SGD-OD mass balance model

To highlight the potential impact of SGD-OD, we developed a simple model using the mass balance of electrons needed to reduce DO in a typical marine ecosystem. During oxygen reduction, molecular oxygen is converted to oxidized substrate and/or water through the gain of four electrons. Thus, to reduce the DO concentration by 100 µmol L^−1^ requires 400 e^−^ L^−1^. These electrons are supplied by the reduced byproducts (electron donors) carried by SGD. For example, to oxidize NH_4_^+^ to NO_3_^−^ requires a loss of 8 electrons (− 8e^−^) and oxidizing 100 µmol L^−1^ of NH_4_^+^ to NO_3_^−^ results in an electron loss of 800 e^−^ L^−1^.

We recognize that Fig. 1 represents a highly simplified version of more complex processes. For example, some electron donors are not oxidized rapidly and thus the true reduction capacity also depends on the timescale and the effects of microorganisms^5^. We assume the inorganic components (primarily H_2_S and NH_4_^+^) oxidize rapidly, within a few days^10^. However, OM is comprised of an array of compounds that degrade at highly variable rates, with less complex OM, such as carbohydrates and proteins, often preferentially utilized over other more complex molecules such as lignin^11^. Labile OM, produced from fresh biomass, consumes DO more typically at a Redfield-type O_2_:C of 0.77^12,13^. In coastal systems OM consumption and biological oxygen demand (BOD) are most often dominated by dissolved organic matter (DOM) and is typically measured as the DO loss that occurs over a 5 to 28 day period^14^. While BOD rates vary significantly, consumption rates greater than 50% of DOC have been found in coastal systems influenced by urbanization^15^. The magnitude of this fraction likely differs depending on the environment. Hereafter, we conservatively assume 15% of the DOC in SGD is oxidized within a few days. This allows us to include DOC in the calculations without putting undue weight on this component. Since CH_4_ in shallow systems evades to the atmosphere within a few hours^15^, and CH_4_ oxidation rates can vary with salinity and temperature^16^, we reduce its potential effect by 60%. While this is a crude approximation, it does allow us to demonstrate the impact of CH_4_ relative to other electron donors given this important by product^17^.

## SGD study sites and results

Fluxes of SGD to estuarine and coastal waters occur episodically. During tidal cycles, SGD usually peaks during falling tide^18^. Evidence for longer time scales is found in changing radium isotope signals^19–22^ and in thermal records in continental shelf sediments^22,23^. The thermal records show short (1–3 day) episodes of strong SGD that are associated with wind patterns that temporarily lower sea level^22,23^. Storm events also produce strong episodic SGD^24–27^. Here, average fluxes and concentrations are used in the model, while recognizing that episodic fluxes may have more serious consequences.

To understand the potential impact of SGD on DO concentration, knowledge of the salty SGD flux is required. One of the most common techniques for constraining salty SGD is the use of radium isotopes^18^. To highlight the potential impacts of SGD-OD on receiving waters, we use site examples where SGD fluxes have been well characterized using radium isotopes and are accompanied by electron donor concentrations. A brief review of the use of radium isotopes in these studies is provided in the Supplemental Information (SI). We use component inventories to describe electron transfers in these shallow environments and to compare across SGD flux studies. An inventory is calculated as the total amount of a component in the water column over a given area. For example, if NH_4_^+^ has a concentration of 35 mmol m^−3^ and is well mixed over a 2.5 m water column, the inventory is 87.5 mmol m^−2^.

### Okatee basin SGD fluxes

The Okatee River and salt marsh are located inland of Hilton Head Island in southeastern South Carolina, USA. This area was the site of the Land Use—Coastal Ecosystem Study (LU-CES) from 1999 to 2005, which studied the hydrography, hydrology, and biogeochemistry of the study area. During 2001–2002, hypoxic conditions (DO < 2 mg L^−1^ or < 64 µmol kg^-1^) were found in 21.5% of the observations in the estuary (n = 20,900)^28,29^. The Okatee River is formed where two small creeks converge in the upper reaches of the study area. The hydrology and biogeochemistry teams focused on the upper Okatee Basin (length = 5.6 km, low tide volume = 590,000 m^3^, tidal prism = 711,000 m^3^). About 80% of the tidal prism that exits the upper Okatee returns essentially unmodified each tidal cycle, resulting in a residence time of 2–4 days^30^.

Monitoring wells were installed perpendicular to the river along two transects in the Basin and radium isotopes, nutrients, carbon, sulfide and Fe^2+^ concentrations quantified. Moore et al.^30^ estimated ^226^Ra fluxes from this system and measured high concentrations of nutrients and carbon in Okatee groundwaters. Porubsky et al.^31^ found significant correlations between NH_4_^+^, PO_4_^3−^, DOC, DIC, DON and ^226^Ra in the groundwater. Fluxes can be calculated if the components are strongly correlated with ^226^Ra and the flux of ^226^Ra from the groundwater is known. We follow Porubsky et al.^31^ who estimated mean fluxes of NH_4_^+^, DOC, and DON into the Basin (Table 2). They did not estimate H_2_S fluxes, so we used the correlation of H_2_S and ^226^Ra (773 µmol dpm^−1^, R^2^ = 0.53) and the ^226^Ra flux of 1.5 × 10^8^ dpm day^−1^ to estimate a mean H_2_S flux of 116 kmol day^−1^ to the Basin. The concentrations of N_2_O, Fe^2+^ and CH_4_ were low and therefore not considered to impact DO consumption significantly.

Areal component fluxes per tidal cycle (mol m^−2^ tc^−1^) were estimated by dividing each component flux by the water area at high and low tide (Table 1). Each of the electron donor fluxes were multiplied by the electron exchange and the results were summed to determine the areal e^−^ flux. The e^−^ flux is dominated by H_2_S, with NH_4_^+^ contributing another 10%. Both DOC and DON are minor contributors. Increasing the fraction of DOC oxidized would have a minor impact at this and other study sites.

**Table 1:** Calculations of the supply of electrons to Okatee River.

Component	Component flux* (kmol tc^−1^)	Areal flux** (mol m^−2^ tc^−1^)	e^−^ supplied*** (mol m^−2^ tc^−1^)	Water depth (m)	DO reduction (mol m^−2^ tc^−1^)	DO reduction (µmol L^−1^ tc^−1^)
High tide						
DOC	23.6	0.033	0.020	2.7	0.005	2
NH_4_^+^	5.9	0.008	0.064	2.7	0.016	6
H_2_S	59.9	0.084	0.672	2.7	0.168	62
DON	0.8	0.001	0.008	2.7	0.002	1
Total			0.76		0.19	71
Low tide						
DOC	23.6	0.116	0.070	1.5	0.017	12
NH_4_^+^	5.9	0.029	0.233	1.5	0.058	39
H_2_S	59.9	0.295	2.361	1.5	0.590	390
DON	0.8	0.004	0.032	1.5	0.008	5
Total			2.69		0.69	450

The areal component flux, electron (e^-^) flux, DO reduction, and volumetric DO reduction are provided on a per tidal cycle basis at both high tide (H) and low tide (L). All values are the mean of all individual sampling events (n = 8) with sufficient data to calculate a groundwater flux.

*Based on a ^226^Ra flux of 1.5 × 10^8^ dpm day^−1^^30,31^

**High tide area = 7.13 × 10^5^ m^2^.

**Low tide area = 2.03 × 10^5^ m^2^.

***We assume 15% of the total DOC flux is oxidized on a 2–4 day timescale.

DO concentrations in the Okatee River range from < 30 to > 300 µm L^−1^^27^. At steady state, the flux of electron donors to the Okatee River could consume 71 to 450 µmol L^−1^ of DO through each tidal cycle. This variability in potential DO reduction is primarily due to the differences in water coverage between high and low tide. SGD is most pronounced during a falling tide^18^ and component fluxes interact with a smaller volume of water over a smaller area. As a result, the reduction potential of DO at low tide increases by more than a factor of 6 (Table 1) and could completely deplete the DO if components are consumed on a tidal timescale of a few hours. It is important to note, however, that SGD initially emerges at the start of ebb tide when there is more water in the system that dilutes the flux until absolute low tide. Furthermore, turbulence generated by exchange of the tidal prism in shallow water, in combination with river discharge, can rapidly entrain atmospheric oxygen into the system. Buzzelli et al.^28^ estimated mean DO utilization rates in the Okatee Basin of 0.15 mol m^−2^ day^−1^. The estimated potential DO reduction at high tide of 0.19 mol m^−2^ tc^−1^ could reduce 2.5 times this DO given the tidal cycle. That the Okatee River experiences only episodic versus pervasive hypoxic conditions suggests that atmospheric exchange in this shallow, turbulent system must explain the difference.

### Three sites near Sapelo Island, GA

Sapelo Island is a ~ 67 km^2^ barrier island situated between mainland Georgia, USA, and the Atlantic Ocean. Its landward coastline is characterized by expansive salt marshes connected to the adjacent estuary through a complex network of tidal channels and creeks. In 2008, transects of PVC monitoring wells were installed across 3 salt marshes around Sapelo Island with 30 cm screened intervals, 1–5 m beneath the marsh platform. These transects stretched from the bank of a tidal creek, across a salt marsh, and up to an adjacent upland area. The sites were designated CI, HN, and PC and are described in detail by Schutte et al.^32^ and in the SI. Samples for radium activity and concentrations of DOC, DON, NH_4_^+^, H_2_S, total Fe, and CH_4_ were collected seasonally from each creek, ocean, and groundwater throughout 2008 and 2009. Schutte et al.^32^ used these data to estimate SGD using a radium mass balance for each sampling period. Further, they used ^228^Ra:^226^Ra mixing curves to identify the wells that tapped the sub-marsh aquifer and to indicate the wells most responsible for exchange with the tidal creek. They used this information to estimate the marsh component of SGD and multiplied SGD by the CH_4_ concentration of the dominant sub-marsh aquifer to determine the SGD-driven CH_4_ flux from the salt marsh. Here, we expand upon this work to determine the SGD-derived flux of other reduced constituents that may contribute to surface water DO demand (Table 2).

**Table 2:** Calculations of the supply of electrons to at the 3 Sapelo Island study sites (CI, HN, and PC).

Site/tide		Comp	Component flux (kmol tc^−1^)	Areal flux* (mol m^−2^ tc^−1^)	e^−^ supplied** (mol m^−2^ tc^−1^)	Water depth (m)	DO reduction (mol m^−2^ tc^−1^)	DO reduction (µmol L^−1^ tc^−1^)
CI	H	DOC	2.23	0.012	0.01	0.65	0.002	3
		NH_4_^+^	1.01	0.006	0.04	0.65	0.011	19
		H_2_S	0.03	0	0	0.65	0	1
		DON	0.18	0.001	0.01	0.65	0.002	3
		Fe	0.05	0	0	0.65	0	0
		CH_4_	0.03	0	0	0.65	0	1
		Total			0.06		0.015	27
	L	DOC	2.23	0.203	0.12	1	0.035	30
		NH_4_^+^	1.01	0.092	0.73	1	0.18	180
		H_2_S	0.03	0.003	0.02	1	0.005	5
		DON	0.18	0.016	0.13	1	0.032	32
		Fe	0.05	0.005	0	1	0.001	1
		CH_4_	0.03	0.003	0.01	1	0.002	2
		Total			1.02		0.25	250
HN	H	DOC	59.9	0.122	0.08	0.62	0.018	25
		NH_4_^+^	0.93	0.002	0.02	0.62	0.004	5
		H_2_S	3.56	0.007	0.06	0.62	0.015	26
		DON	0.91	0.002	0.01	0.62	0.004	5
		Fe	0.01	0	0	0.62	0	0
		CH_4_	0.06	0	0	0.62	0	0
		Total			0.17		0.041	61
	L	DOC	59.9	2.220	1.33	1	0.33	330
		NH_4_^+^	0.93	0.035	0.28	1	0.069	69
		H_2_S	3.56	0.132	1.05	1	0.26	260
		DON	0.91	0.034	0.27	1	0.067	67
		Fe	0.01	0	0	1	0	0
		CH_4_	0.06	0.002	0.01	1	0.002	2
		Total			2.94		0.73	730
PC	H	DOC	25.4	0.079	0.05	0.81	0.012	14
		NH_4_^+^	0.8	0.003	0.02	0.81	0.005	6
		H_2_S	2.05	0.006	0.05	0.81	0.013	27
		DON	0.82	0.003	0.02	0.81	0.005	6
		Fe	0.04	0.024	0.02	0.81	0.006	7
		CH_4_	7.82	0	0	0.81	0	0
		Total			0.16		0.041	60
	L	DOC	25.4	0.94	0.56	1	0.141	140
		NH_4_^+^	0.8	0.03	0.24	1	0.059	59
		H_2_S	2.05	0.076	0.61	1	0.152	150
		DON	0.82	0.03	0.24	1	0.060	60
		Fe	0.04	0.29	0.29	1	0.072	72
		CH_4_	7.82	0.001	0.01	1	0.001	1
		Total			1.95		0.490	490

The areal component flux, electron (e^−^) flux, DO reduction, and volumetric DO reduction are provided on a per tidal cycle basis at both high tide (H) and low tide (L). All values are the mean of all individual sampling events with sufficient data to calculate a groundwater flux at each study site (CI, n = 8, HN, n = 4, PC, n = 6).

*High tide area for CI = 1.8 × 10^5^ m^2^, HN = 4.9 × 10^5^ m^2^, PC = 3.2 × 10^5^ m^2^.

*Low tide area CI = 1.1 × 10^4^ m^2^, HN = 2.7 × 10^4^ m^2^, PC = 2.7 × 10^4^ m^2^.

**We assume 15% of the total DOC flux and 40% of the CH_4_ is oxidized on a 2–4 day timescale.

High tide total DO demand across all sites and time periods ranged from 4 to 110 µmol L^−1^ tc^−1^ with a median value of 34 µmol L^−1^ tc^−1^. Low tide total DO demand was an order of magnitude higher, ranging from 48 to 1500 µmol L^−1^ tc^−1^ with a median value of 335 µmol L^−1^ tc^−1^. The higher demand at low tide again reflects the order of magnitude lower volume of surface water in the estuary relative to high tide. There was between-site variability in total DO demand, with site HN having the highest demand and site CI having the lowest. However, there were no seasonal patterns in total DO demand either within or across sites.

The groundwater components that contributed most significantly to total DO demand were highly variable both spatially (CI, PC, and HN) and temporally (PC and HN). For example, at site HN, DOC contributed 41–45% of the total DO demand followed by H_2_S (36–43%). At site CI, NH_4_ was dominant, contributing 70–72% of the total DO demand. At site PC H_2_S dominated (31–45%) DO demand followed by Fe^2+^ (12–15%).

### Mississippi coast

Mississippi Sound is an estuary located in the northern Gulf of Mexico along the coasts of Mississippi and Alabama, USA. Hypoxic events and fish kills occur frequently along the Mississippi coast, often in the western-most section^33–36^. A time series of five stations on the western Sound beaches were conducted between July 2017 and November 2019, where surface water and groundwater samples were sampled for radium, NH_4_^+^, DOC, CH_4_, and Fe^2+^^37^. Groundwater H_2_S samples were collected in January 2023 from each of the five stations. A mixing model using ^228^Ra was constructed to determine the SGD flux into the Sound at each station per month, ranging from < 0 (i.e., seawater intrusion) to 62 cm^3^ cm^−2^ day^−1^, with an average SGD flux of 9.9 cm^3^ cm^−2^ day^−1^ during summer and fall^37^.

**Figure 2:**
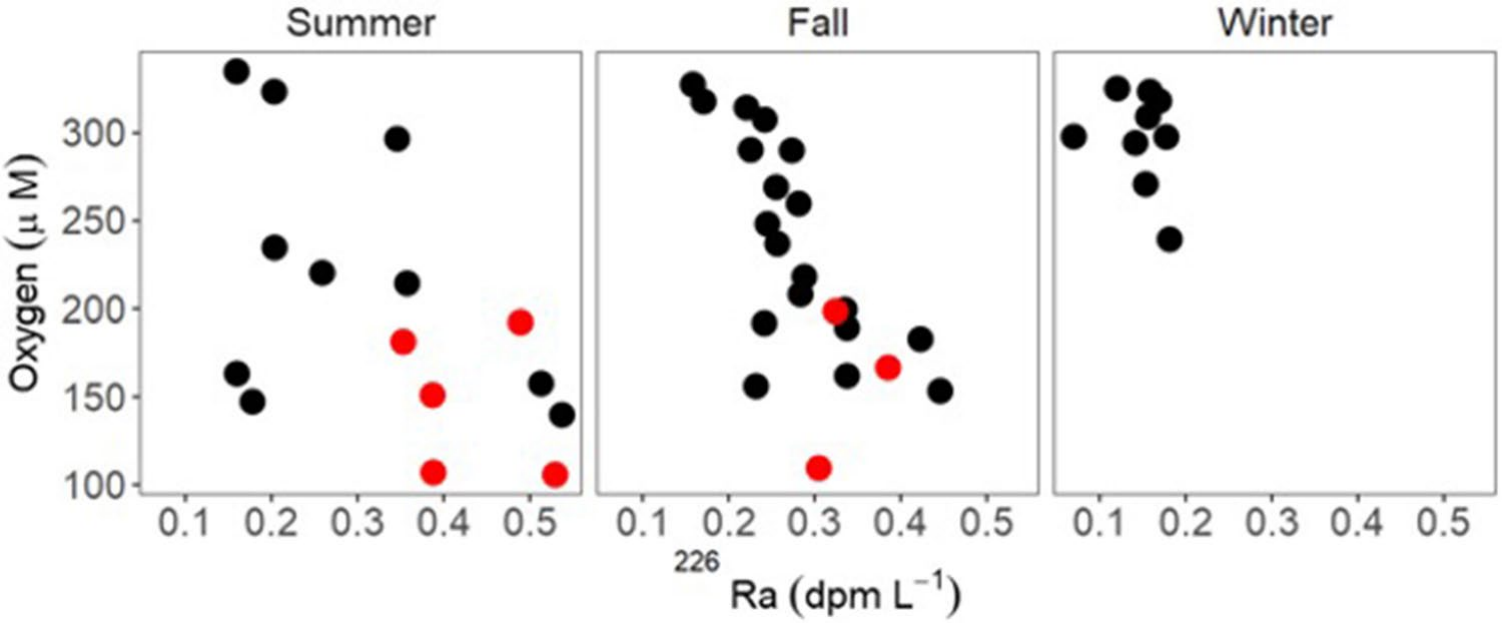
Dissolved oxygen versus ^226^Ra at near shore sites in the western Mississippi Sound. Despite the shallow depth of sample collection (1 m), low concentrations of DO are associated with high ^226^Ra activities along the Mississippi coast. Red circles denote samples collected during verified fish kills at the different sites.

Lowest DO values averaged 207 µmol O_2_ L^−1^ during the summer and early fall. Based on temperature data, the average saturation of the water was 259 µmol O_2_ L^−1^, a reduction of 52 µmol O_2_ L^−1^, or 0.05 mol DO m^−2^ in a 1 m water column. This requires 0.2 mol e^−^ m^−2^. In surface waters, there is a trend of decreasing DO with increasing ^226^Ra (Fig. 2) that qualitatively suggests that SGD is contributing to DO depletion.

Unlike the Okatee Basin and Sapelo Island, tidal influences were not directly measured and likely play a smaller role in SGD discharge in this system. The e^-^ load to coastal waters was therefore calculated on a daily basis using groundwater endmember concentrations collected from the 5 stations, the SGD flux, and the electron flux based on the number of electrons exchanged (Table 3). The e^−^ supply from SGD was 0.63 mol m^−2^ day^−1^^38^. H_2_S clearly dominated the electron balance. On average, the total DO demand from SGD was 160 µmol DO L^−1^ day^−1^, though the average DO concentration remained above 200 µmol L^−1^. Continual aeration of these shallow waters appears to prevent DO concentrations from declining further. Atmospheric invasion of DO into the water column was calculated from the actual DO concentration, potential saturated DO, and the wind speed^39,40^. The average atmospheric invasion of DO into the water column was 373 µmol DO L^−1^ day^−1^ of O_2_ into the shallow water column^39,40^. Turbulence could further increase aeration. During the summer and fall when fish kills were observed, the oxygen invasion exceeded 200 µmol L^−1^ day^−1^. Therefore, oxygen resupply in this shallow environment offset the DO reduction due to SGD. Three fish kills were verified during this study even though DO at our sites did not approach hypoxic conditions, perhaps due to the high abundance of hydrogen sulfide (see “Discussion”). Hypoxic conditions further offshore may have also led to the fish kills at these times.

**Table 3:** Calculation of the electron supply from SGD to the Mississippi Sound.

Component	GW endmember (µM)	Areal flux (mol m^−2^ day^−1^)*	e^−^ supplied (mol m^−2^ day^−1^)**	DO reduction (µmol L^−1^ d^−1^)
H_2_S	577	0.057	0.46	120
DOC	363	0.036	0.02	5
NH_4_^+^	112	0.011	0.09	22
DON	71	0.007	0.06	14
Fe^2+^	8	0.001	0.00	< 1
CH_4_	4	0.000	0.00	< 1
Total			0.63	160

*Based on SGD flux of 9.9 cm^3^ cm^−2^ day^−1^.

**We assume 15% of the total DOC flux and 40% of the CH_4_ is oxidized on a 2–4 day timescale.

### Offshore South Carolina coast

Data from a monitoring station on the northern South Carolina, USA coast at Myrtle Beach reveal episodic periods of hypoxic conditions in bottom waters in the late summer. The station is maintained by Coastal Carolina University at the end of Apache pier, which extends 250 m into the ocean. Surface and bottom waters are monitored continuously. The real-time and archived data from Apache pier are available online: http://hydrometcloud.com/hydrometcloud/index.jsp**.**

Radium samples collected from the end of Apache pier in mid-August 2012 contain the highest ^226^Ra and ^228^Ra activities ever measured in the South Atlantic Bight, exceeding measurements earlier in the summer by about one order of magnitude^21^. After eliminating other sources of radium, Peterson et al.^21^ concluded the enrichment must be due to SGD from aquifers on the continental shelf. They used the ^228^Ra/^226^Ra activity ratio (AR) in the samples to identify the source as an aquifer tapped by monitoring wells A and R located about 18 km offshore^21^. Using the average radium activities that had been measured in the wells, they determined that SGD from this aquifer could support an inventory of 1.7 m^3^ m^−2^ of SGD in the study area.

Peterson et al.^21^ found average bottom water DO values changed at the end of Apache pier (4.5 to 6.5 m depth depending on tide) from 175 µmol DO L^−1^ (n = 8) prior to the discharge event on 4 August, to 102 µmol DO L^−1^ (n = 15) on 16–17 August, a reduction of 73 µmol DO L^−1^. Taking the bottom water thickness as 2 m based on temperature profiles, this translates into a reduction of 0.15 mol DO m^−2^, requiring 0.6 mol e^−^ m^−2^. Peterson et al.^21^ concluded that if the SGD contained no DO, dilution alone could explain the observed reduction in DO.

The composition of water in wells A and R was measured from 1999 to 2013. From these data (see SI), the average concentration (in µmol L^−1^) of each potential electron donor was used with the SGD inventory measured in August 2012 to calculate an e^−^ inventory of 3.53 mol m^−2^ (Table 4). The inventory is six times higher than that needed to reduce bottom water DO concentrations by 75 µmol L^−1^. Because the site is close to the surf zone located only 250 m from shore, waves and turbulence at the air-sea interface must constantly resupply DO through atmospheric exchange.

**Table 4:** SGD components and their effects on DO off the coast of South Carolina measured during two SGD events.

Component	GW endmember (µmol L^−1^)	Component inventory (mol m^−2^)	e^−^ supplied*** (mol m^−2^)	DO reduction (mol m^−2^)	Water depth (m)	DO reduction (µmol L^−1^)
Aug. 2012						
DOC	362	0.093*	0.371	0.09	2	46
NH_4_^+^	135	0.231*	1.847	0.46	2	230
H_2_S	52	0.089*	0.711	0.18	2	89
DON	44	0.075*	0.602	0.15	2	75
Total			3.53	0.88		440
Aug. 2019						
DOC	362	0.014**	0.056	0.01	8.9	2
NH_4_^+^	135	0.035**	0.281	0.07	8.9	8
H_2_S	52	0.014**	0.108	0.03	8.9	3
DON	44	0.011**	0.092	0.02	8.9	3
Total			0.54	0.13		16

*Based on an SGD inventory of 1.7 m^3^ m^−2^.

**Based on an SGD inventory of 0.26 m^3^ m^−2^.

***We assume 15% of the total DOC flux is oxidized on a 2–4 day timescale.

**Figure 3:**
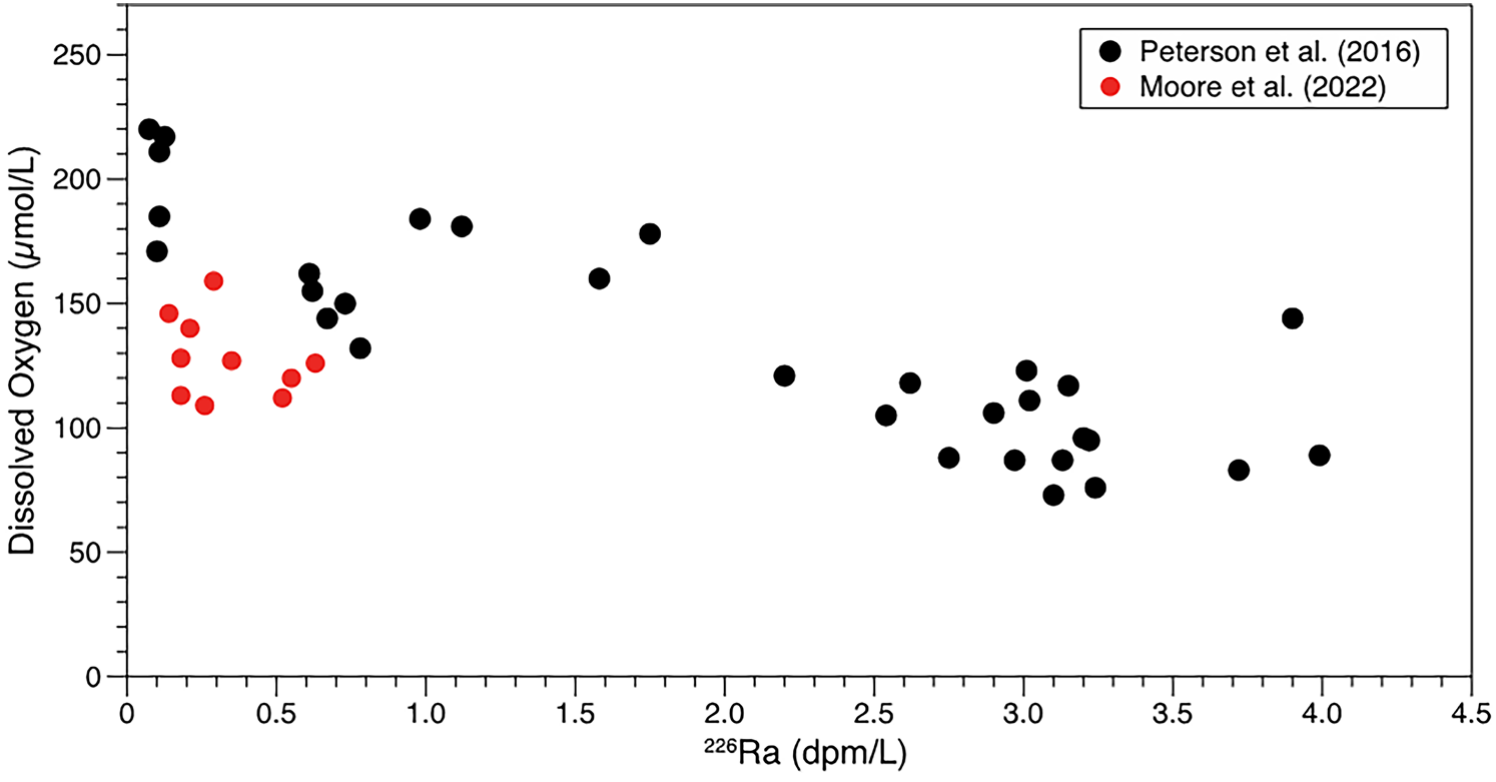
Comparison of August 2012 (black circles) and August 2019 (red circles) DO and ^226^Ra off the coast of South Carolina. Data from^21,22^.

Another episodic offshore SGD event was sampled in August 2019 (Table 4) with samples spanning about ~ 170 km from Apache Pier to 10–20 km offshore of Charleston, SC^22^. This episodic event was predicted based on the wind field in late July 2019. Bottom waters were depleted in DO and enriched in radium, but not to the extent as measured at Apache pier in 2012. Figure 3 compares the 2012 and 2019 DO and Ra data.

In August 2019 the bottom waters off Charleston contained an average of 124 µmol DO L^−1^. Unlike August 2012, the DO content of the bottom water immediately prior to the SGD event is unknown. We therefore use data from May 2019 when bottom water DO averaged 137 µmol L^−1^^22^. This implies a decrease of 13 µmol DO L^−1^ between May and August and a total e^-^ demand of 0.46 e^−^ m^−2^ for the 8.9 m bottom water column. Based on the radium isotope composition, wells A and R were again identified as the source of the radium enrichment and an SGD inventory of 0.26 m^3^ m^−2^ was estimated^22^. This translates into an e^−^ supply of 0.54 mol e^−^ m^−2^, more than sufficient to account for the reduction in DO observed over the time period (Table 4).

## Discussion

Hypoxia is recognized to be widespread, exerting significant biological stress in coastal ecosystems^1^. For example, coastal zooplankton and fish suffer several deleterious effects, including reduced prey capture efficiency, growth and reproductive potential, and even death^41^. Hypoxic zones further diminish and compress habitats, by making deeper, cooler waters unavailable in the summer^42^. Indeed, ecosystems exposed to long periods of hypoxia are characterized by low annual secondary production and minimal to no benthic fauna. Hypoxic zones in the Baltic Sea and Chesapeake Bay are estimated to have caused a loss in secondary production of ~ 6000 to 10,000 MT C annually; for the Gulf of Mexico, this loss may be as high as 17,000 MTC annually^1^. Increasing hypoxia also influences benthic biogeochemistry, both directly and indirectly. With the reduction in benthic fauna, sediment bioturbation is reduced, exacerbating the impacts of low DO on pore water chemistry, limiting oxygen-requiring reactions, *e.g.*, coupled nitrification/denitrification, and stimulating loss of metals and phosphorus from sediments into the overlying water column.

### Top-down vs bottom-up hypoxia mechanism

The classic top-down mechanism for generating coastal hypoxia first involves the supply of abundant nutrients to coastal waters, which increases biological productivity. The high productivity increases OM sedimentation to the seabed where this OM is oxidized by DO and drives hypoxia in stratified bottom waters that cannot receive new DO by mixing with surface or offshore waters. SGD can play a top-down role in DO depletion by supplying excess nutrients. Many studies have concluded that SGD nutrient supply is often greater than the supply from local rivers^18,43–48^. Alternately, SGD also plays an immediate bottom-up role in decreasing DO concentrations by supplying electron donors to bottom waters that reduce DO directly.

Numerous papers suggest a link between SGD and low DO concentrations in estuarine and coastal waters^49–54^. Few papers link the reduced components in SGD directly to oxygen consumption in these systems. Peterson et al.^21^ demonstrated that simple dilution of bottom water with minimal DO SGD input could explain the DO reduction at Apache Pier in 2011. Guo et al.^55^ measured SGD fluxes to the Changjiang (Yangtze) River estuary in China based on a ^222^Rn mass balance model and found that SGD fluxes were higher in the summer when hypoxic conditions prevailed in the estuary and lower in the winter when hypoxic conditions were absent. They suggested SGD contributed to hypoxic conditions by either a top-down or bottom-up mechanism. Sanial et al.^9^ applied a multi-tracer model to evaluate chemical mass balances in bottom waters along the coastline south of the Mississippi Sound and concluded that a common mechanism must supply Ra isotopes, Ba, and Si. After eliminating other sources, they concluded SGD must supply these components, driving oxidation of reduced SGD species (NH_4_^+^, H_2_S, CH_4_, DOC) and consuming DO in the process, thus promoting the development of seasonal hypoxia. Inverse correlations between the SGD proxy ^228^Ra and DO in bottom waters further substantiated their hypothesis.

Here, we provide a mechanistic link between SGD and DO reduction and present supporting data from a variety of disparate coastal environments. These results further offer insights into when and where SGD-OD is most likely to influence coastal hypoxia. Estuaries experience quite different potential DO depletions at low and high tide due to extreme changes in water depth and areal extent. In the studies highlighted here, potential depletions at high tide ranged from 27 to 71 µmol L^−1^. During low tide, potential depletions increased by an order of magnitude and would have resulted in anoxic conditions if no aeration occurred (Table 5).

**Table 5:** Comparisons of the estuarine and coastal systems.

Site	Potential DO reduction high tide (µmol L^−1^ tc^−1^)	Potential DO reduction low tide (µmol L^−1^ tc^−1^)
Estuaries		
Okatee	71	450
CI	27	260
HN	61	740
PC	61	490

Coasts	e^−^ available (mol m^−2^)	Potential DO reduction (µmol L^−1^ day^−1^)
SC coast (2012)	3.53	440
SC coast (2019)	0.54	15
Miss sound	0.63	160

### Effects of H_2_S besides DO reduction

Specific components of SGD, such as hydrogen sulfide, which at pH < 7 is the most prominent sulfide species^56^, may also have direct detrimental effects irrespective of DO. The 96-h LC_50_ H_2_S concentration for marine fish is 1.5 to 15 µmol L^−1^^57^. For example, throughout the year, water in the Okatee estuary has a pH ~ 7^31^, meaning about 50% of dissolved sulfide is H_2_S (the HS^-^ ion is much less toxic than H_2_S)^57^. This water usually contains some measurable H_2_S with median concentrations much higher from April to September (hot) compared to October through March (cool) (Table 6). Here, the total dissolved sulfide concentration was divided by 2 to estimate the H_2_S concentration. The number of samples having > 1 µmol L^−1^ during the hot period was 11 out of 13, while in the cool period only 3 of 8 samples exceeded 1 µmol L^−1^. A sample collected on 26 April 2002 was omitted, as it had 145 µmol sulfide L^−1^ and is viewed as an outlier. The average hot period H_2_S concentrations are above the lower limit of 1.5 µmol L^−1^ for 96-h LC_50_. These H_2_S concentrations will kill some organisms and likely stress many more.

**Table 6:** Cool–Hot weather comparison of H_2_S concentrations in the Okatee estuary.

Dates 2002–2005	Number samples	Average H_2_S (µmol L^−1^)	Median H_2_S (µmol L^−1^)	Number > 1 µmol L^–1^
Cool October–March	8	1.3	0.5	3
Hot April–September	13	1.7	2.1	11
All year	21	1.5	1.3	14

Millero et al.^58^ measured the half-life of H_2_S in air-saturated seawater at pH 8.0 to be 26 ± 9 h. However, Luther et al.^59^ demonstrated that abiotic H_2_S oxidation rates yielded half-lives of hundreds of hours when conducted under oxic, clean, sterile conditions. Yet, when the sulfide oxidation rates were measured in the presence of chemolithotrophic microbes, light, and oxygen, they increased three orders of magnitude. The implication of these findings is that the residence time of H_2_S will largely be determined by the population size and activity of microbial communities rather than by chemical oxidants alone.

We estimated an SGD flux of 0.084 mol H_2_S m^−2^ to the Okatee estuary during a tidal cycle (Table 1). Assuming a 3-day residence time for the water, a 2.7 m mean depth and an H_2_S concentration equal to 50% of the total dissolved sulfide, this SGD flux would produce an initial concentration of 93 µmol H_2_S L^−1^ before oxidation. With 93 µmol H_2_S L^−1^ as the initial concentration, a yearly median of 1.3 µmol H_2_S L^−1^ as the final concentration, and assuming first order kinetics, we determined a H_2_S half-life of 0.5 days. This is about 1 tidal cycle during which the initial H_2_S supply is exported with the tidal prism down the estuary. Given the half-life, only about 40% of this H_2_S is present in the return flow as about 80% of the tidal prism returns to the upper estuary without significant mixing during each tidal cycle^30^. Thus, only about 10% of the H_2_S that is introduced in the upper estuary is supplied to the lower estuary. Of course, there may be additional SGD further downstream.

## Conclusions

Electron donors supplied by SGD significantly reduce the DO concentrations in estuarine and coastal waters. Of the systems considered, sulfide (HS^−^/H_2_S) was the most dominant electron donor in about half the cases. Ammonia (NH_4_^+^), DOC or DON were dominant in the rest. Although the potential to deplete DO below hypoxic conditions was certainly present in many cases, reductions were counterbalanced by invasion of oxygen from the atmosphere in the shallower coastal systems. Our use of average fluxes and concentrations may have further underestimated the DO potential during periods of high SGD.

The subterranean estuary is expanding because of sea level rise and mining of freshwater aquifers^4^. This expansion increases the contact between sulfate and OM that has not been in contact with seawater for thousands of years. The byproducts of sulfate–OM reactions enrich the subterranean estuary in a variety of electron donors. As these electron donors are transported to estuarine and coastal waters by SGD, the potential for DO reduction is very likely to increase. More extensive studies of SGD-OD should therefore be conducted in stratified waters to better understand the role of SGD in direct DO consumption in coastal ecosystems.

### Supplementary Information

Supplementary Information 1.

Supplementary Information 2.

## Data Availability

All of the data used in this paper has been published in papers referenced in the text or is presented in the Supplementary Information.

## References

[1] Diaz RJ, Rosenberg R (2008). Spreading dead zones and consequences for marine ecosystems. Science.

[2] Rabalais NN (2010). Dynamics and distribution of natural and human-caused hypoxia. Biogeosciences.

[3] Moore WS (1999). The subterranean estuary: A reaction zone of ground water and sea water. Mar. Chem..

[4] Moore WS, Joye SB (2021). Saltwater intrusion and submarine groundwater discharge: Acceleration of biogeochemical reactions in changing coastal aquifers. Front. Earth Sci..

[5] Ruiz-González C, Rodellas V, Garcia-Orellana J (2021). The microbial dimension of submarine groundwater discharge: Current challenges and future directions. FEMS Microbiol. Rev..

[6] Moore WS (2010). The effect of submarine groundwater discharge on the ocean. Ann. Rev. Mar. Sci..

[7] Paytan A (2006). Submarine groundwater discharge: An important source of new inorganic nitrogen to coral reef ecosystems. Limnol. Oceanogr..

[8] Cardenas MB (2020). Submarine groundwater and vent discharge in a volcanic area associated with coastal acidification. Geophys. Res. Lett..

[9] Sanial V, Moore WS, Shiller AM (2021). Does a bottom-up mechanism promote hypoxia in the Mississippi Bight?. Mar. Chem..

[10] Joye SB, Hollibaugh JT (1995). Influence of sulfide inhibition of nitrification on nitrogen regeneration in sediments. Science.

[11] Baldock JA, Masiello CA, Gélinas Y, Hedges JI (2004). Cycling and composition of organic matter in terrestrial and marine ecosystems. Mar. Chem..

[12] Moreno, A. R. *et al.* Latitudinal gradient in the respiration quotient and the implications for ocean oxygen availability. 10.1073/pnas.2004986117/-/DCSupplemental. 10.1073/pnas.2004986117 PMC7502759 32868433

[13] Johnson, K. S. *Simultaneous Measurements of Nitrate, Oxygen, and Carbon Dioxide on Oceanographic Moorings: Observing the Redfield Ratio in Real Time*. http://www.mbari.org.

[14] *APHA Standard Methods for Examination of Water and Wastewater*. (2005).

[15] Petrone KC, Fellman JB, Hood E, Donn MJ, Grierson PF (2011). The origin and function of dissolved organic matter in agro-urban coastal streams. J. Geophys. Res..

[16] de Angelis MA, Scranton MI (1993). Fate of methane in the Hudson river and estuary. Glob. Biogeochem. Cycles.

[17] Roberts HM, Shiller AM (2015). Determination of dissolved methane in natural waters using headspace analysis with cavity ring-down spectroscopy. Anal. Chim. Acta.

[18] Santos IR (2021). Submarine groundwater discharge impacts on coastal nutrient biogeochemistry. Nat. Rev. Earth Environ..

[19] Moore WS, Shaw TJ (1998). Chemical signals from submarine fluid advection onto the continental shelf. J. Geophys. Res. Oceans.

[20] Moore WS (2007). Seasonal distribution and flux of radium isotopes on the southeastern U.S. continental shelf. J. Geophys. Res. Oceans.

[21] Peterson RN (2016). A new perspective on coastal hypoxia: The role of saline groundwater. Mar. Chem..

[22] Moore WS, Vincent J, Pickney JL, Wilson AM (2022). Predicted episode of submarine groundwater discharge onto the South Carolina, USA, Continental Shelf and its effect on dissolved oxygen. Geophys. Res. Lett..

[23] George C (2020). A new mechanism for submarine groundwater discharge from continental shelves. Water Resour. Res..

[24] Hu C, Muller-Karger FE, Swarzenski PW (2006). Hurricanes, submarine groundwater discharge, and Florida’s red tides. Geophys. Res. Lett..

[25] Moore WS (2002). Thermal evidence of water exchange through a coastal aquifer: Implications for nutrient fluxes. Geophys. Res. Lett..

[26] Moore WS, Wilson AM (2005). Advective flow through the upper continental shelf driven by storms, buoyancy, and submarine groundwater discharge. Earth Planet Sci. Lett..

[27] Wilson AM, Moore WS, Joye SB, Anderson JL, Schutte CA (2011). Storm-driven groundwater flow in a salt marsh. Water Resour. Res..

[28] Buzzelli C, Holland AF, Sanger DM, Conrads PC (2007). Hydrographic characterization of two tidal creeks with implications for watershed land use, flushing times, and benthic production. Estuaries Coasts.

[29] Kleppel, G. S. & Devoe, M. R. *South Atlantic Bight Land Use-Coastal Ecosystem Study (LU-CES. 2002–2003 Annual Progress Report*. www.scseagrant.org (2004).

[30] Moore WS, Blanton JO, Joye SB (2006). Estimates of flushing times, submarine groundwater discharge, and nutrient fluxes to Okatee Estuary, South Carolina. J. Geophys. Res. Oceans.

[31] Porubsky WP, Weston NB, Moore WS, Ruppel C, Joye SB (2014). Dynamics of submarine groundwater discharge and associated fluxes of dissolved nutrients, carbon, and trace gases to the coastal zone (Okatee River estuary, South Carolina). Geochim. Cosmochim. Acta.

[32] Schutte CA, Moore WS, Wilson AM, Joye SB (2020). Groundwater-driven methane export reduces salt marsh blue carbon potential. Glob. Biogeochem. Cycles.

[33] Gunter G, Lyles CH (1979). Localized plankton blooms and jubilees on the Gulf Coast. Gulf Res. Rep..

[34] Overstreet, R. M. & Hawkins, W. E. Diseases and mortalities of fishes and other animals in the Gulf of Mexico. in* Habitats and Biota of the Gulf of Mexico: Before the Deepwater Horizon Oil Spill.* (Springer, 2017). 10.1007/978-1-4939-3456-0_6.

[35] Engle VD, Summers JK, Macauley JM (1999). Dissolved oxygen conditions in northern Gulf of Mexico Estuaries. Environ. Monit. Assess..

[36] Mississippi Department of Marine Resources (MDMR). *Jubilee Occurring in Mississippi Sound; Seafood Safe to Eat, But People Should Use Caution*. (2017).

[37] Moody, A. *Evaluating the Impact of Submarine Groundwater Discharge on Nutrients and Trace Elements in Coastal Systems: The Examples of the Tuckean Swamp (Australia) and the Mississippi Sound (USA)*. (The University of Southern Mississippi, 2022).

[38] Matoušů A, Osudar R, Šimek K, Bussmann I (2017). Methane distribution and methane oxidation in the water column of the Elbe estuary, Germany. Aquat. Sci..

[39] Garcia HE, Gordon LI (1992). Oxygen solubility in seawater: Better fitting equations. Limnol. Oceanogr..

[40] Wanninkhof R (2014). Relationship between wind speed and gas exchange over the ocean revisited. Limnol. Oceanogr. Methods.

[41] Roman MR, Brandt SB, Houde ED, Pierson JJ (2019). Interactive effects of Hypoxia and temperature on coastal pelagic zooplankton and fish. Front. Mar. Sci..

[42] Breitburg, D. *Effects of Hypoxia, and the Balance Between Enrichment, on Coastal Fishes and Fisheries Hypoxia and*. www.dlesapeake.net (2002).

[43] Rodellas V (2015). Submarine groundwater discharge as a major source of nutrients to the Mediterranean Sea. Proc. Natl. Acad. Sci. USA.

[44] Wang G (2015). Net subterranean estuarine export fluxes of dissolved inorganic C, N, P, Si, and total alkalinity into the Jiulong River estuary, China. Geochim. Cosmochim. Acta.

[45] Crotwell, A. M. & Moore, W. S. *Nutrient and Radium Fluxes from Submarine Groundwater Discharge to Port Royal Sound, South Carolina*. *Aquatic Geochemistry*, vol. 9 (2003).

[46] Ji T (2013). Nutrient inputs to a Lagoon through submarine groundwater discharge: The case of Laoye Lagoon, Hainan, China. J. Mar. Syst..

[47] Niencheski LFH, Windom HL, Moore WS, Jahnke RA (2007). Submarine groundwater discharge of nutrients to the ocean along a coastal lagoon barrier, Southern Brazil. Mar. Chem..

[48] Valiela, I. *et al. Transport of Groundwater-Borne Nutrients from Watersheds and Their Effects on Coastal Waters*, vol. 10. https://www.jstor.org/stable/1468685?seq=1&cid=pdf (1990).

[49] Hwang DW, Lee YW, Kim G (2005). Large submarine groundwater discharge and benthic eutrophication in Bangdu Bay on volcanic Jeju Island, Korea. Limnol. Oceanogr..

[50] Lee YW, Kim G (2007). Linking groundwater-borne nutrients and dinoflagellate red-tide outbreaks in the southern sea of Korea using a Ra tracer. Estuar. Coast Shelf Sci..

[51] Su N (2014). Natural radon and radium isotopes for assessing groundwater discharge into Little Lagoon, AL: Implications for harmful algal blooms. Estuaries Coasts.

[52] Luo X, Jiao JJ, Moore WS, Lee CM (2014). Submarine groundwater discharge estimation in an urbanized embayment in Hong Kong via short-lived radium isotopes and its implication of nutrient loadings and primary production. Mar. Pollut. Bull..

[53] Luo X (2018). Significant chemical fluxes from natural terrestrial groundwater rival anthropogenic and fluvial input in a large-river deltaic estuary. Water Res..

[54] Montiel D, Lamore A, Stewart J, Dimova N (2019). Is submarine groundwater discharge (SGD) important for the historical fish kills and harmful algal bloom events of Mobile Bay?. Estuaries Coasts.

[55] Guo X (2020). Does submarine groundwater discharge contribute to summer hypoxia in the Changjiang (Yangtze) River Estuary?. Sci. Total Environ..

[56] Holmer M, Hasler-Sheetal H (2014). Sulfide intrusion in seagrasses assessed by stable sulfur isotopes: A synthesis of current results. Front. Mar. Sci..

[57] Boyd, C. & Tucker, C. *Handbook for Aquaculture Water Quality*. (2014).

[58] Mlllero FJ, Hublnger S, Fernandez M, Garnett S (1987). Oxidation of H_2_S in seawater as a function of temperature, pH, and ionic strength. Environ. Sci. Technol.

[59] Luther GW (2011). Thermodynamics and kinetics of sulfide oxidation by oxygen: A look at inorganically controlled reactions and biologically mediated processes in the environment. Front. Microbiol..

