# Supplementary material for: SGD-OD: investigating the potential oxygen demand of submarine groundwater discharge in coastal systems
Source: Sci Rep. 2024 Apr 22;14:9249. doi: 10.1038/s41598-024-59229-7 (PMC11035578; doi:10.1038/s41598-024-59229-7)
Supplement: Supplementary file 1 — Supplementary Information 1. [file 41598_2024_59229_MOESM1_ESM.docx]

**SGD-OD: Investigating the Potential Oxygen Demand of Submarine Groundwater Discharge in Coastal Systems: Supplemental Information**

**Use of Ra isotopes in SGD studies**

Both chemical and nuclear properties of radium make this element an excellent tracer for submarine groundwater discharge (SGD). Chemically the Ra^2+^ ion adsorbs to particles in low ionic strength, neutral pH solutions. Thus, in freshwater systems a large fraction of radium is usually particle-bound, resulting in low activities of dissolved radium in most freshwater aquifers. This behavior changes if the ionic strength increases. In systems containing seawater, the particle-bound Ra exchanges primarily with Ca^2+^, Mg^2+^, K^+^, and Na^+^ ions, allowing Ra^2+^ to escape to solution. High radium activities are usually found in salty aquifers.

Radium exists as four naturally occurring isotopes: ^226^Ra (half-life = 1600 yrs), ^228^Ra (half-life = 5.7 yrs), ^224^Ra (half-life = 3.6 days), and ^223^Ra (half-life = 11 days). These are often called the Radium Quartet. They each derive from decay of a thorium isotope. Because thorium is almost completely particle bound in most environments, it provides a constant source of radium isotopes that may desorb from terrestrial and marine sediments. Because of the different half-lives, each Ra isotope regenerates at different rates after it desorbs. After a salty aquifer is flushed of radium, ^224^Ra and ^223^Ra regenerate on a time scale of days, while ^228^Ra and ^226^Ra require years to centuries to regenerate from parent decay. Their presence indicates supply from a more distant source or from remineralization and release from aquifer solids. After their release by SGD, ^224^Ra and ^223^Ra decay on a time scale of days, allowing age estimates of when SGD supplied the radium.

To use Ra to estimate SGD fluxes, we must first quantify other processes that affect Ra in coastal waters including inputs from rivers, release from riverine particles, regeneration and release from sediments, removal by radioactive decay, adsorption on particles and/or coprecipitation with barite. For ^228^Ra and ^226^Ra, decay, regeneration and adsorption/coprecipitation can be considered minimal in most systems.

Here, we explore the potential of SGD-OD along the US southeastern and Gulf coasts using studies that have relied on radium-based SGD models and use (1) correlations between ^226^Ra and components in multiple wells, (2) single aquifers as primary sources, and (3) multiple aquifers as primary sources to reduced elements and compound fluxes in coastal ecosystems. Our results confirm that SGD can play a significant role in low DO throughout these ecosystems.

We primarily use ^226^Ra whose half-life of 1600 years negates regeneration and decay on the daily to monthly timescales considered here. The riverine ^226^Ra flux (both dissolved and release from riverine particles) along the SE US coast has been well characterized^[1]^, contributing less than 10% of the ^226^Ra flux to coastal waters. After surface sediments lose ^226^Ra, hundreds of years are required to regenerate significant new activities, meaning we can neglect this source. We have not found evidence of ^226^Ra removal on short timescales; in the open ocean the timescale for removal is on the order of 500 years^[2]^. Thus, SGD is the primary source of ^226^Ra enrichment at the sites addressed in this paper. The primary loss is mixing with more dilute offshore waters. To determine the ^226^Ra flux, we first measure its inventory in the designated study area and then use physical oceanography or short-lived radium isotopes, ^224^Ra and ^223^Ra, to characterize residence times. The residence time tells how much of the inventory must be replaced each day, i.e., the ^226^Ra flux out of the system. After making a small river correction, if necessary, the flux out of the system is balanced by the flux into the system if steady state conditions apply on the timescale of interest.

In some cases, there are clear relationships between radium and other components in coastal aquifers. For example, Porubsky, et al.^[3]^ demonstrated strong correlations of DIC, DOC, NH_4_^+^, PO_4_^3-^, CH_4_, HS^-^, and N_2_O with ^226^Ra activities in monitoring wells at Okatee, SC. Knowing the ^226^Ra flux and the relationship of ^226^Ra and each component in SGD, Porubsky, et al.^[3]^ estimated fluxes of these components to the Okatee River. The average ^226^Ra flux was 1.5 x 10^8^ dpm d^-1^ and the average NH_4_^+^/^226^Ra ratio in the monitoring wells was 75.5 µmol/dpm, resulting in a NH_4_^+^ flux of 11.3 kmol d^-1^. Similar relationships have been observed in offshore monitoring wells in Long Bay, SC, where Moore et al.^4^ found total dissolved N/^226^Ra = 26.4 µmol/dpm (R^2^ = 0.877) and total dissolved P/^226^Ra = 1.3 µmol/dpm (R^2^ = 0.813) in November 1999. From these observations they calculated fluxes of TDN and TDP to the coastal ocean. Surprisingly, subsequent sampling campaigns to these wells did not reveal such strong correlations. In the Pearl River delta, China, Luo et al.^[5]^ found a correlation between the depletion of sulfate and ^226^Ra activity in monitoring wells on the shore of the Pearl River to estimate an SGD NH_4_^+^ flux of 2.4 x 10^9^ mol N y^-1^ to the Pearl River.

If there is not a strong relationship between ^226^Ra and other components in monitoring wells, we must identify which wells are tapping aquifers most likely to be supplying the ^226^Ra. One key to this identification is the ^228^Ra/^226^Ra activity ratio (AR) of the excess radium in the water column. These two isotopes are generated on different timescales by decay of parent isotopes in different decay chains. The ^238^U decay chain produces ^226^Ra; the ^232^Th decay chain produces ^228^Ra. Although both parents are ubiquitous in the environment, uranium has higher concentrations in carbonates and phosphorites; thorium is more concentrated in clays and silts^[6]^. These separations produce differences in the ^228^Ra/^226^Ra AR in SGD from different aquifers. In some cases, radium enrichments in coastal waters have a distinct ^228^Ra/^226^Ra AR which may closely match the AR in one or more monitoring wells. For example, Moore et al.^[7]^ found Ra-enriched bottom water off the coast of SC in August 2019 had a ^228^Ra/^226^Ra AR that followed a tight linear trend with slope = 1.4 and R^2^ = 0.99. This trend intersected the AR of the Gulf Stream further offshore, implying the Ra enrichment was caused by mixing between a high Ra source and dilute offshore waters. Samples from two monitoring wells on the continental shelf were highly enriched in radium and closely matched the measured ^228^Ra/^226^Ra AR, implying the aquifer tapped by these wells was the source of the enriched radium and other components to the coastal waters. In other cases, a single source cannot be identified, implying the sources of the radium and other components are multiple aquifers^[8,9]^. Schutte et al.^[10]^ developed a mixing model utilizing measurements from several aquifers to identify the fraction of radium from each system necessary to explain the enrichments in nearby creeks. This approach is detailed in section 3b**.**

**Table SI-1.** Groundwater fluxes and high and low tide estuary area and volume estimates for the 3 study sites around Sapelo Island, Georgia. Values are either single best estimates or the mean ± the standard deviation of the number of measurements listed in column named “n”.

| **Site** | **Groundwater flux**  **(10^6^ L tc^-1^)** | **n** | **Estuary area**  **High tide**  **(10^5^ m^2^)** | **Estuary area**  **Low tide**  **(10^4^ m^2^)** | **Estuary volume**  **High tide**  **(10^8^ L)** | **Estuary volume**  **Low tide**  **(L)** |
| --- | --- | --- | --- | --- | --- | --- |
| CI | 2.06 ± 0.85 | 8 | 1.8 | 1.1 | 1.16 ± 0.34 | 1.10 x 10^7^ |
| HN | 16.3 ±13.9 | 4 | 4.9 | 2.7 | 3.05 ± 1.05 | 2.70 x 10^7^ |
| PC | 28.1 ± 19.8 | 6 | 3.2 | 2.7 | 2.59 ± 1.09 | 2.70 x 10^7^ |

**Table SI-2.** Radium and electron donor composition of wells A and R from 2019 to 2013. Radium activities in dpm L^-1^; electron donors in µmol L^-1^.

| **Well** | **Date** | **Salinity** | **^226^Ra** | **^228^Ra** | **NH_4_** | **DON** | **DOC** | **H_2_S** |
| --- | --- | --- | --- | --- | --- | --- | --- | --- |
| well A | 20-Jul-99 |  | 6.05 | 9.93 |  |  |  |  |
| well A | 24-Jul-99 |  | 6.04 | 8.86 |  |  |  |  |
| well A | 8-Sep-99 | 35 | 5.98 | 9.15 |  |  |  |  |
| well A | 10-Sep-99 | 35.15 | 6.69 | 8.53 |  |  |  |  |
| well A | 9-Nov-99 | 35.2 | 5.41 | 8.71 | 93.2 | 42.8 |  |  |
| well A | 10-Nov-99 | 35.2 | 4.83 | 6.72 | 91.2 | 41.7 |  |  |
| well A | 14-Nov-99 |  | 4.65 | 7.23 |  |  |  |  |
| well A | 14-Nov-99 |  | 5.89 | 7.76 |  |  |  |  |
| well A | 26-May-00 | 35.3 | 7.86 | 11.48 |  |  |  |  |
| well A | 26-May-00 | 35.2 | 4.54 | 5.54 |  |  |  |  |
| well A | 2-Jun-00 | 35.3 | 4.69 | 7.04 |  |  |  |  |
| well A | 2-Jun-00 | 35.1 | 4.15 | 6.25 |  |  |  |  |
| well A | 4-Jun-00 | 35.1 | 3.89 | 6.15 |  |  |  |  |
| well A | 4-Jun-00 | 35.2 | 4.62 | 7.00 |  |  |  |  |
| well A | 31-Aug-00 | 35.09 | 5.24 | 7.38 |  |  |  |  |
| well A | 31-Aug-00 | 35.05 | 7.50 | 10.27 |  |  |  |  |
| well A | 4-Sep-00 | 35.02 | 5.13 | 6.70 |  |  |  |  |
| well A | 4-Nov-00 |  | 5.62 | 6.97 |  |  |  |  |
| well A | 4-Nov-00 |  | 4.85 | 5.97 |  |  |  |  |
| well A | 8-Nov-00 |  | 4.60 | 6.08 |  |  |  |  |
| well A | 8-Nov-00 |  |  |  |  |  |  |  |
| well A | 7-Feb-01 | 35.9 | 2.75 | 3.64 | 38 | 37.7 |  |  |
| well A | 7-Feb-01 | 35.6 | 2.91 | 4.87 | 44 | 39.7 |  |  |
| well A | 7-Feb-01 | 35.6 | 3.76 | 5.01 | 44 | 44.5 |  |  |
| well A | 7-Feb-01 | 35.9 | 3.19 | 4.27 | 40 | 37.3 |  |  |
| well A | 23-Aug-01 | 35.2 | 3.52 | 4.82 | 7 |  | 190 |  |
| well A | 9-Jul-02 | 34.4 | 6.31 | 8.14 | 101 | 32.3 | 199 |  |
| well A | 9-Jul-02 | 34.4 | 6.47 | 8.36 | 96 | 29.2 | 186 |  |
| well A | 31-Jul-03 | 34.7 | 6.06 | 8.45 | 84 |  | 344 | 11.5 |
| well A | 31-Jul-03 | 34.8 | 6.32 | 8.54 | 89 |  | 343 | 9.5 |
| well A | 25-Oct-04 | 34.8 | 6.67 | 8.87 | 129 |  | 244 | 109.7 |
| well A | 25-Oct-04 | 35.0 | 5.31 | 7.06 | 119 |  | 256 | 114.2 |
| well A | 9-Sep-13 | 35.15 | 5.44 | 8.23 | 115 |  |  |  |
| well R | 1-Sep-00 |  | 10.04 | 12.47 |  |  |  |  |
| well R | 4-Nov-00 |  | 3.33 | 4.62 |  |  |  |  |
| well R | 23-Aug-01 | 34.9 | 6.17 | 8.45 | 69 |  | 555 |  |
| well R | 9-Jul-02 | 34 | 9.33 | 12.34 | 408 | 86.6 | 565 |  |
| well R | 31-Jul-03 | 34.7 | 7.34 | 8.69 | 182 |  |  | 14.4 |
| well R | 26-Oct-04 | 34.1 | 8.48 | 12.56 | 563 |  | 740 | 50.3 |
| well R | 10-Sep-13 | 34.91 | 8.92 | 11.89 | 250 |  |  |  |
| **Average A & R** |  | **35.03** | **5.65** | **7.82** | **135** | **44** | **362** | **51.6** |

**Determination of concentrations of dissolved electron donors**

Samples were filtered (0.2 µm) and stored (4°C) prior to analysis (within days to a week). Ammonium (NH_4_) samples were immediately fixed with 4 phenol and concentrations were quantified using the phenol-hypochlorite technique^[11]^ usually within 1–2 days. Concentrations of nitrate plus nitrite (=NOx) were determined using standard protocols for groundwater samples^[12]^ or a Lachat autoanalyzer (QuikChem 8000) for surface water samples. Samples for the determination of total dissolved nitrogen (TDN) were processed using high temperature catalytic oxidation^[12,13]^. DON was estimated by difference (DON = TDN – NO_x_ – NH_4_). Hydrogen sulfide (H_2_S) was quantified by colorimetry^[14]^. Dissolved organic carbon (DOC) concentrations were determined using a Shimadzu TOC5000^[12,15]^.

**References**

1. Moore, W. S. & Shaw, T. J. Fluxes and behavior of radium isotopes, barium, and uranium in seven Southeastern US rivers and estuaries. *Mar Chem* **108**, 236–254 (2008).

2. Moore, W. S. & Dymond, J. Correlation of 210 Pb removal with organic carbon fluxes in the Pacific Ocean. *Nature* **331**, 339–341 (1988).

3. Porubsky, W. P., Weston, N. B., Moore, W. S., Ruppel, C. & Joye, S. B. Dynamics of submarine groundwater discharge and associated fluxes of dissolved nutrients, carbon, and trace gases to the coastal zone (Okatee River estuary, South Carolina). *Geochim Cosmochim Acta* **131**, 81–97 (2014).

4. Moore, W. S. *et al.* Thermal evidence of water exchange through a coastal aquifer: Implications for nutrient fluxes. *Geophys Res Lett* **29**, (2002).

5. Luo, X. *et al.* Significant chemical fluxes from natural terrestrial groundwater rival anthropogenic and fluvial input in a large-river deltaic estuary. *Water Res* **144**, 603–615 (2018).

6. Moore, W. S. *Sources and fluxes of submarine groundwater discharge delineated by radium isotopes*. *Biogeochemistry* vol. 66 (2003).

7. Moore, W. S., Vincent, J., Pickney, J. L. & Wilson, A. M. Predicted Episode of Submarine Groundwater Discharge onto the South Carolina, USA, Continental Shelf and Its Effect on Dissolved Oxygen. *Geophys Res Lett* **49**, (2022).

8. Young, M. B. *et al.* Characterizing sources of groundwater to a tropical coastal lagoon in a karstic area using radium isotopes and water chemistry. *Mar Chem* **109**, 377–394 (2008).

9. Lewis, B. L. *et al.* Short-term and interannual variability of redox-sensitive chemical parameters in hypoxic/anoxic bottom waters of the Chesapeake Bay. *Mar Chem* **105**, 296–308 (2007).

10. Schutte, C. A., Moore, W. S., Wilson, A. M. & Joye, S. B. Groundwater-Driven Methane Export Reduces Salt Marsh Blue Carbon Potential. *Global Biogeochem Cycles* **34**, (2020).

11. Solórzano, L. Determination Of Ammonia In Natural Waters By The Phenolhypochlorite Method 1 *Limnology and Oceanography* vol. 14 799–801 Preprint at https://doi.org/10.4319/lo.1969.14.5.0799 (1969).

12. Weston, N. B. *et al.* Porewater stoichiometry of terminal metabolic products, sulfate, and dissolved organic carbon and nitrogen in estuarine intertidal creek-bank sediments. *Biogeochemistry* **77**, 375–408 (2006).

13. Álvarez-Salgado, X. A. & Miller, A. E. J. Simultaneous determination of dissolved organic carbon and total dissolved nitrogen in seawater by high temperature catalytic oxidation: conditions for precise shipboard measurements. *Mar Chem* **62**, 325–333 (1998).

14. CLINE, J. D. Spectrophotometric Determination of Hydrogen Sulfide In Natural Waters. *Limnology and Oceanography* vol. 14 454–458 Preprint at https://doi.org/10.4319/lo.1969.14.3.0454 (1969).

15. Bouillon, S. *et al.* Inorganic and organic carbon biogeochemistry in the Gautami Godavari estuary (Andhra Pradesh, India) during pre-monsoon: The local impact of extensive mangrove forests. *Global Biogeochem Cycles* **17**, (2003).
